# Evaluation of Synovial Calprotectin by Using a Lateral Flow Test for the Diagnosis of Prosthetic Joint Infections

**DOI:** 10.3390/diagnostics13040741

**Published:** 2023-02-15

**Authors:** Marta Bottagisio, Marco Viganò, Antonio Pellegrini, Nicola Logoluso, Luigi Zagra, Andrea Prina, Laura de Girolamo, Elena De Vecchi

**Affiliations:** 1 IRCCS Istituto Ortopedico Galeazzi, Laboratory of Clinical Chemistry and Microbiology, 20161 Milan, Italy; 2 IRCCS Istituto Ortopedico Galeazzi, Orthopedics Biotechnology Lab, 20161 Milan, Italy; 3 IRCCS Istituto Ortopedico Galeazzi, Centre for Reconstructive Surgery and Osteoarticular Infections C.R.I.O. Unit, 20161 Milan, Italy; 4 IRCCS Istituto Ortopedico Galeazzi, Hip Department, 20161 Milan, Italy

**Keywords:** prosthetic joint infection, calprotectin, synovial fluid, biomarkers, diagnosis of PJI, orthopedics

## Abstract

The analysis of synovial fluid is a crucial step in the diagnosis of prosthetic joint infections (PJIs). Recently several studies illustrated the efficacy of synovial calprotectin in supporting the diagnosis of PJI. In this study, synovial calprotectin was analyzed by a commercial stool test to explore whether it might accurately predict PJIs. The synovial fluids of 55 patients were analyzed and calprotectin levels were compared to other synovial biomarkers of PJI. Of the 55 synovial flu-ids, 12 patients were diagnosed with PJI and 43 with an aseptic failure of the implant. Specificity, sensitivity, and AUC of calprotectin resulted in 0.944, 0.80, and 0.852 (95%CI: 0.971–1.00), respectively, with a set threshold of 529.5 µg/g. Calprotectin had a statistically relevant correlation with the synovial leucocyte counts (rs = 0.69, *p* < 0.001) and the percentage of synovial neutrophils (rs = 0.61, *p* < 0.001). From this analysis, it can be concluded that synovial calprotectin is a valuable biomarker that correlates with other established indicators of local infection, and the use of a commercial lateral flow stool test could be a cost-effective strategy delivering rapid and reliable results and supporting the diagnostic process of PJI.

## 1. Introduction

Prosthetic joint infection (PJI) represents a serious complication in orthopedic prosthetic surgery that requires specific and accurate microbiological diagnosis to ensure the best clinical management of this adverse event [1]. 

Microbiological tests largely contribute to the diagnosis of PJI, allowing the isolation and identification of the etiological agent and the definition of its antibiotic susceptibility profile [2]. An early and indicative investigation can be performed intraoperatively with the analysis of the synovial fluid; however, a definitive diagnosis can be only achieved in the postoperative phase by the microbiological culture of periprosthetic tissues, prosthetic material, and/or synovial fluid aimed at retrieving the etiological cause [3,4,5]. Indeed, the isolation and identification of the pathogen through the culture of explanted specimens are essential to the diagnosis of PJI; however, the entire culture process requires at least 2–5 days for aerobic bacteria and up to 7 days for anaerobes [6]. 

In this context, the extemporaneous analysis of the synovial fluid (e.g., leukocyte esterase, leukocyte count) might guide the surgical intervention, providing a faster response and suggesting the presence of an infection before culture results. In recent years, several studies reported the promising results of leukocyte esterase and alpha defensin testing in the diagnosis of PJI [7,8,9,10,11]. However, the detection of esterase may produce false-positive results in the case of metal-to-metal reactions and inflammatory non-infectious conditions, while the high costs for the determination of alpha defensin levels limit its use in daily laboratory practice [11,12,13,14]. There are few studies currently available in the literature evaluating other inflammatory markers, such as calprotectin at the synovial level [15,16]. Although promising results have been published to date [17,18], there is still the need to find a standardized, rapid, and cost-effective strategy to quantify the concentration of calprotectin, to overcome the limits set by the expensive lateral flow test designed for synovial fluids currently available and/or the complicated and time- consuming laboratory methodologies. Indeed, the study of a faster screening tool that allows for reducing the turnaround time of microbiological examinations could lead to a significant improvement in the management of PJIs, reducing costs for the healthcare system and improving the timing of diagnosis.

In this context, the use of a commercial lateral flow point-of-care (POC) stool test could be a fast and reliable strategy. These assays show an accuracy comparable to ELISA measurements and the straightforward protocol is very practical. However, these com-mercial POC tests are designed for the measurement of calprotectin levels in solid and liquid stool samples, generating the need to validate its diagnostic value on different biological matrices. 

Hence, in this study, calprotectin levels in synovial fluids of patients who underwent revision surgery of the prosthetic implant were analyzed by means of a commercial lateral flow point-of-care stool test (Quantum Blue^®^ fCAL, BÜHLMANN) to explore whether it might predict PJIs and, therefore, being a promising tool for the fast and reliable diagnosis of this complication.

## 2. Materials and Methods

### 2.1. Study Design

Fifty-five patients were enrolled in this study. Written, informed consent was obtained from all the volunteers participating in the study according to the Ethical Review Board of IRCCS San Raffaele Hospital (protocol no. CE:146/int/2018; date of approval 11 October 2018).

According to the approved protocol, only patients diagnosed with a failure of the prosthetic implant undergoing a revision procedure at the IRCCS Galeazzi Orthopedic Institute were enrolled after expressing their consent. Therefore, arthrocentesis was aseptically performed in the operating room prior arthrotomy and the joint aspirate was immediately sent to the Laboratory of Clinical Chemistry and Microbiology and stored at −80 °C until analysis. 

Once the surgical procedure was completed, samples of periprosthetic tissues, synovial fluid, and the implant itself collected intraoperatively were sent to the laboratory for microbiological culture as part of the standard diagnostic routine. Aliquots of eluate obtained after treatment with 0.1% dithiothreitol (DTT, Sigma-Aldrich, Milan, Italy) of tissues and prostheses were plated on chocolate, MacConkey, mannitol salt, and Sabouraud agar and inoculated into brain heart infusion (BHI) (all BioMeriéux, Marcy-l’Étoile, France) and thioglycollate broths (Thermo Fisher Diagnostics, Rodano, Italy) and incubated until a visible microbiological growth or up to 15 days [19]. Besides that, analysis of serum (i.e., C-reactive protein (CRP) and erythrocyte sedimentation rate (ESR)) and synovial fluid (i.e., esterase, leukocyte count, percentage of synovial neutrophils) were carried out for all the enrolled patients, before the surgical procedure begins to avoid any postoperative peaks. 

Subjects were then classified as infected or non-infected according to the major and minor criteria proposed by the Musculoskeletal Infection Society (MSIS) and Consensus Meeting of Philadelphia. In particular, the isolation of microorganisms by culture from at least two periprosthetic samples or evidence of a sinus tract communicating with the joint space were considered major criteria, while criteria based on hematological tests (CRP and ESR) and synovial fluid analyses (esterase, leukocyte count, percentage of synovial neutrophils) were considered as minor [3,4].

### 2.2. Blood and Synovial Fluid Analyses

The quantification of CRP concentration in serum was measured on an Atellica^®^ CH Analyzer (Siemens Healthineers, Germany), whereas ESR levels were determined by means of the Alifax Test1 automated analyzer (Alifax S.p.A., Polverara, Italy) on EDTA blood samples. 

Leukocyte esterase in synovial fluid was detected by means of a colorimetric strip (Dirui Industrial Co., Ltd., Changchun, China) and graded according to the enzymatic reaction. Briefly, synovial fluids were centrifuged at 3000 rpm for 10 min to limit possible interference caused by the presence of corpuscles. Then, one drop of the synovial fluid supernatant was placed on the leukocyte esterase pad of the strip and read after a two-minute incubation. The color change to purple indicated the presence of the enzyme and, according to the color intensity, samples were classified as POS+, POS++, and POS+++. Traces or absence of color were considered negative.

Finally, leukocyte and neutrophils counts were performed on synovial fluid collected into sterile tubes containing K3 EDTA as an anticoagulant (Becton Dickinson, NJ, USA) by means of an automated cell counter (Sysmex XE XN2100 Haematology Auto-mated System, Sysmex Partec Italia, (Cornaredo, Milan, Italy) within 30 min from the sample arrival using a dedicated channel (Body Fluids, Cornaredo, Milan, Italy).

### 2.3. Calprotectin Lateral Flow Test

Synovial levels of calprotectin were detected by means of the lateral flow test Quantum Blue^®^ fCAL Extended (BÜHLMANN, Schönenbuch, Switzerland), following the protocol for liquid samples. Briefly, 10 μL of synovial fluids were pipetted in the CALEX^®^ Cap device, vortexed for 30 s, and incubated for 10 min. Thereafter, 60 µL of the diluted extract was then loaded on the test cassette and, after 12 min, automatically scanned by the Quantum Blue^®^ Reader. The Quantum Blue^®^ Reader revealed the quantification of the test (T) line as well as of the control (C) lines, which was used as a validity check of the assay. The standard range of the obtained results was 30–1000 µg/g.

### 2.4. Statistical Analysis

Analysis was performed using R software v 4.1.1 (R Core Team, Wien, Austria) and package “pROC” [20]. According to the result of the normality test (Shapiro–Wilk), the levels of serum (CRP and ESR) and synovial (calprotectin, leukocyte count, and differential) markers were compared between septic and aseptic failures using the Mann-Whitney test. Since the esterase test provided categorical results (negative, POS+, and POS++), the comparison between the two groups was performed using the chi-squared test for trend. Receiver operator characteristics curves were designed using the culture test as the gold standard to evaluate the area under the curve (AUC) of each method (with the DeLong method for confidence-interval calculation). Thresholds were calculated to maximize Youden’s index; sensitivity and specificity were reported, as well as the positive likelihood ratio (LR+) and negative likelihood ratio (LR−). One-to-one Pearson’s correlation coefficients were calculated for each pair of methods. Levels of calprotectin in subjects showing different results at the esterase test were compared using the Kruskal–Wallis test with the Dunn post hoc test. A *p*-value < 0.05 was considered statistically significant.

## 3. Results

The outcomes of 55 patients undergoing an implant revision procedure at the IRCCS Galeazzi Orthopedic Institute were evaluated. In particular, 43 subjects underwent a replacement intervention due to an aseptic failure of the implant, while the remaining 12 were operated due to an infection. The demographic characteristics of the analyzed patients are shown in Table 1.

### Evaluation of Serum and Synovial Biomarkers of PJI

**Figure 1:**
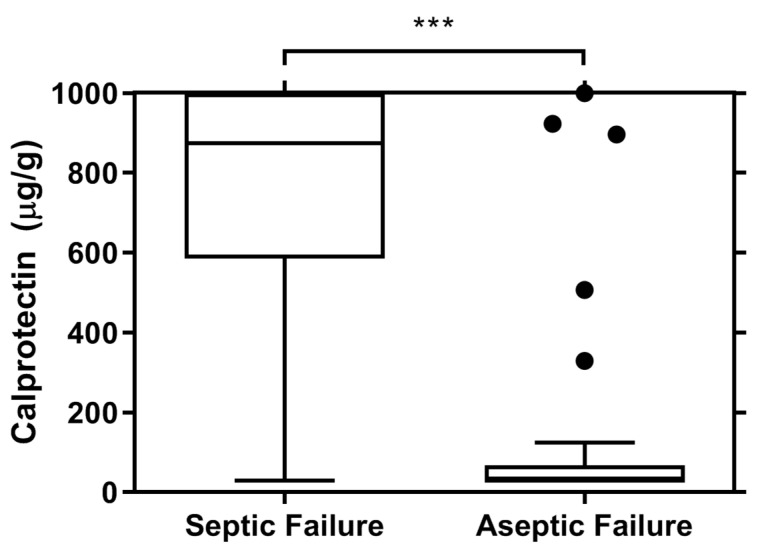
Calprotectin levels distribution among septic and aseptic cases. Statistical significance: *** p < 0.001.

The median synovial calprotectin level was significantly different between the infected cases (median = 874 μg/g, Q1-Q3: 652.5–1000; IQR 347.5) and the aseptic failure cases (median = 30 μg/g, Q1-Q3: 30–66.25; IQR 36.25) for a *p* < 0.001 (Figure 1). Setting the cut-off at 529.5 µg/g, the calprotectin measurement gave rise to 3 false positive and 2 false negative results.

All the serum and synovial biomarkers evaluated were significantly higher in infected than in non-infected patients (as reported in Table 2). 

Furthermore, the sensitivity, specificity, LR+/−, and test threshold were calculated, along with the AUC, to define the overall accuracy and performance of the tested PJI biomarkers (Table 3). 

**Figure 2:**
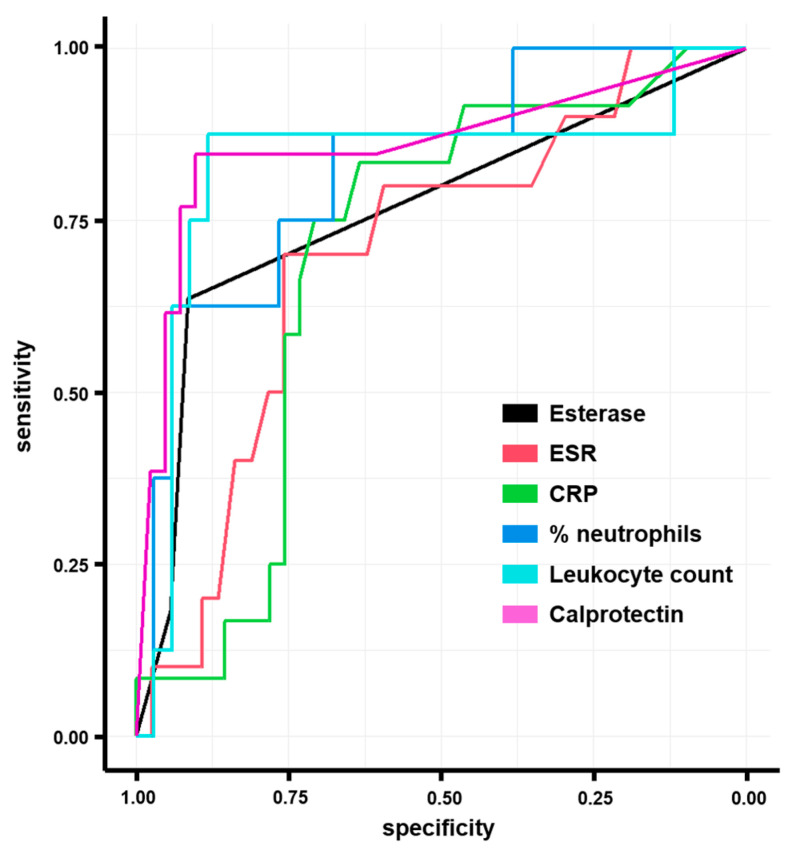
ROC curves for the tests based on serum and synovial markers.

In particular, the detection of synovial calprotectin showed a sensitivity of the lateral flow test of 80% and specificity of 94%, and the ROC curve analysis resulted in an AUC of 0.852 (95% CI 0.971–1.000) for the diagnosis of PJI. The calculated threshold was 529.5 μg/g (Figure 2).

**Figure 3:**
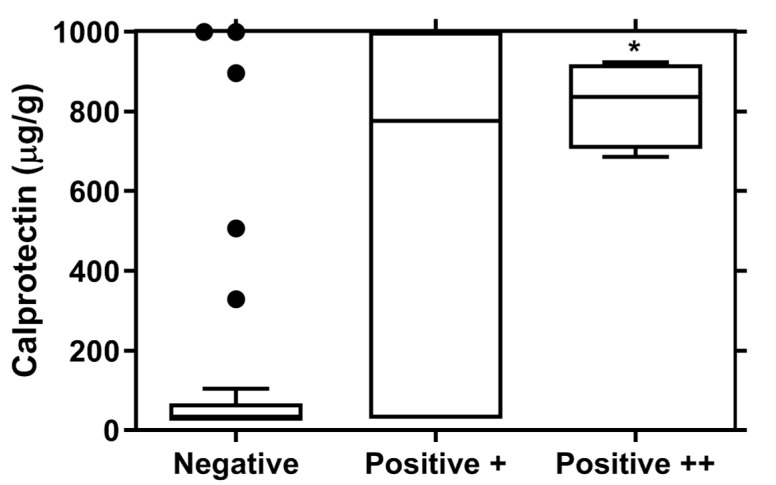
Calprotectin levels distribution in subjects with different results at the esterase test. POS+ and POS++ patients showed significantly higher levels of calprotectin compared to negative subjects. * *p* < 0.05 vs. negative subjects.

The linear relationship between calprotectin values and the other PJI biomarkers is displayed in Table 4. Calprotectin levels were significantly different depending on the result of the esterase test (Figure 3).

**Figure 4:**
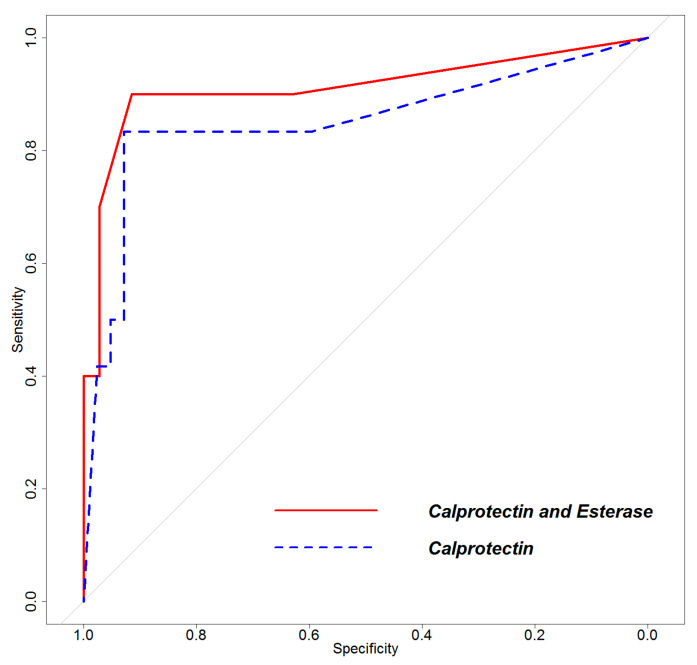
ROC curves obtained by combining the calprotectin and esterase tests or by using the calprotectin test alone.

Considering ROC with two predictors, a test based on the combination of calprotectin and esterase provided the highest AUC compared to other combinations (AUC: 0.911, 95%CI: 0.777–1). The combination of calprotectin and leukocyte count showed AUC = 0.849, while AUC was 0.899, 0.891, and 0.829 for calprotectin and percentage of neutrophils, CRP and ESR, respectively. Figure 4 shows the improvement in the AUC of ROC curves obtained by combining results from calprotectin and esterase tests compared to the ROC curve of the calprotectin test only.

## 4. Discussion

The search for reliable, unbiased biomarkers able to predict the presence of an infection is a vital clinical need in the management of PJI. In this context, synovial fluid markers play an important role in the diagnosis of PJI. The levels of cytokines such as interleukins (i.e., IL-6, IL-1-β, IL-8, IL-17) and other mediators of inflammations (e.g., TNF-α, IFN-γ, etc.) have been investigated in depth, because of their release from white blood cells within the infected joint [21,22]. However, their local modulation might be influenced by other chronic conditions other than infections (e.g., rheumatoid arthritis, inflammatory arthritis, etc.), resulting in a low specificity [23,24]. Similarly, the impact of the systemic inflammatory status in the variation of CRP and ESR values is an important issue that needs to be taken into account when evaluating the PJI biomarker panel [25]. Furthermore, the increase in CRP and ESR is likely the result of an acute infective event rather than of a chronic infection mediated by low-virulence bacteria, weakening the predicting value of serum biomarkers alone in this clinical scenario [26]. Hence, to limit the false positive cases and to guide the interpretation of the clinical picture, local markers related to the expression of antimicrobial functions have been investigated. In particular, synovial leukocyte count and leukocyte esterase, as well as the percentage of neutrophils, showed encouraging results not only in the present investigation but also reported in the literature [27]. Lately, also the predictive value of synovial calprotectin has been brought to light, offering the opportunity to fill the lack of information with new evaluations aimed at testing the potential of this known biomarker in a different diagnostic field [17]. 

In clinical practice, the ease of use and the brief turnaround time have a great impact on the choice of the diagnostic tool; therefore, a lateral flow test that provides the result in 20 min is usually preferred to an ELISA test, even at the expense of sensitivity [28]. The only calprotectin lateral flow test specifically designed for synovial fluid analysis available on the market is currently excessively expensive; the price is not competitive and compatible with the routine use being not a standalone diagnostic method. To overcome this economic challenge, in the present study, the synovial calprotectin levels were evaluated by means of a commercial lateral flow stool test to explore the feasibility of this cost-effective strategy for the diagnosis of PJI on a different biological matrix. To adapt the procedure specifically developed for use with stools to the synovial fluid, we applied the protocol for liquid feces; only 10 μL of synovial fluids are dosed, not interfering with other diagnostic tests, which may require larger volumes of sample.

Taking into consideration the cut-off values set by the CE-IVD labeled Point of Care test designed for synovial fluids (>50 mg/L high, 14–50 mg/L moderate, and <14 mg/L low risk) [29], the Quantum Blue^®^ fCAL extended assay was preferred to Quantum Blue^®^ sCAL test for the quantification of calprotectin in serum (both BÜHLMANN, assay range: 30–1000 µg/g and 0.42–10 µg/mL, respectively). Moreover, it has been demonstrated that Quantum Blue^®^ lateral flow test for the detection of fecal calprotectin can be considered a solid alternative to ELISA assay [30]. 

The obtained data demonstrated a high diagnostic value of calprotectin, comparable to, if not higher than the other serum and synovial markers of PJI. Indeed, the specificity of calprotectin was in line with that observed for synovial esterase and percentage of neutrophils (0.90, 0.91, and 0.94, respectively), but the sensitivity resulted in being higher (0.84, 0.63, and 0.62 respectively). 

The calprotectin test showed false negative results in two patients where a calprotectin level <30 μg/g was detected in the presence of an elevated esterase (POS+) and four false positive results, likely the consequence of an inflammatory condition, occurred in aseptic patients with altered serum CRP and ESR values. 

Lastly, the association of esterase and calprotectin has proved to be the most powerful tool for predicting the presence of a periprosthetic infection, where the synergy of the combined tests reached an AUC of 0.96.

Despite the elevated specificity and sensitivity demonstrated by calprotectin as a biomarker of PJI, the number of evaluations performed in the present study is relatively restricted, emphasizing the need to widen the sample size in future investigations to comprehend also patients with different comorbidities that might interfere with the evaluation (rheumatoid arthritis, diabetes, etc.). Furthermore, it should also be investigated the impact of the freeze-thaw procedure on the measurement of calprotectin concentration. Indeed, a possible drawback of this study could be the storage of the samples at −80 °C before testing because the freeze-thaw process may result in leukocyte cell lysis and a consequent increase in calprotectin levels. Therefore, the cut-off values recommended by the available test designed for synovial fluids might not necessarily be appropriate for our thawed samples, which are in accordance with other data obtained by using the Quantum Blue^®^ test [31].

## 5. Conclusions

From this analysis, it can be concluded that synovial calprotectin is a sensitive and specific biomarker that correlates with other established indicators of local infection. This new biomarker could be easily used in clinical settings due to its high sensitivity and specificity, supporting the results obtained by the evaluation of other biomarkers [17,32]. 

Furthermore, the use of a commercial lateral flow stool test could be a cost-effective promising option delivering rapid and reliable results, supporting the diagnostic process of PJI. The results obtained by applying the protocol for liquid samples of the Quantum Blue^®^ fCAL extended assay, indeed, were predictive of the presence of a PJI. 

For all the aforementioned reasons, this study supports the use of the Quantum Blue^®^ fCAL extended assay for the measurement of synovial calprotectin as a diagnostic criterion for PJI in the near future.

## 6. Patents

Written informed consent was obtained from all the volunteers participating in the study according to the Ethical Review Board of the IRCCS San Raffaele Hospital (protocol no. CE:146/int/2018; date of approval 11 October 2018).

## Figures and Tables

**Table 1:** Demographic information of analyzed subjects and their distribution per year. The percentage reported refers to the number of cases per year. Data referring to patients’ age are reported as mean ± standard deviation.

Patients		Septic Failure	Aseptic Failure
Sex	F	8 (67%)	25 (58%)
	M	4 (33%)	18 (42%)
Age	F	73 ± 7	75 ± 8
	M	63 ± 24	67 ± 12
	all	70 ± 15	72 ± 10
Age group	25–50	1 (8%)	3 (7%)
	51–80	9 (75%)	31 (72%)
	≥81	2 (17%)	9 (21%)

**Table 2:** Median and IQR values of the serum and synovial markers of PJI.

Biomarker		Septic Failure	Aseptic Failure	*p*-Value
Serum CRP (mg/L)	median	1.17	0.30	0.03
	IQR	0.66 (1.065–1.73)	0.765 (0.085–0.85)	
Serum ESR (mm/h)	median	40	19	0.024
	IQR	13 (33–46)	19.5 (11.25–30.75)	
Synovial leukocyte esterase (score)	Negative	3	34	0.002
	POS+	5	1	
	POS++	3	1	
Synovial leukocyte (×10^3^ CFU/μL)	median	24.23	0.87	0.003
	IQR	16.9 (14.19–31.09)	1.67 (0.50–2.17)	
Synovial percentage of neutrophils	median	91.1	38.9	<0.001
	IQR	7.3 (85.95–93.25)	24.9 (22.85–47.75)	
Synovial calprotectin (μg/g)	median	874	30	<0.001
	IQR	347.5 (652.5–1000)	36.25 (30–66.25)	

**Table 3:** Sensitivity, specificity, LR+/−, test threshold, and the AUC of tested PJI biomarkers.

Biomarker	Specificity	Sensitivity	Threshold	AUC	95% CI	LR+	LR−
Calprotectin	0.944	0.800	529.5	0.852	0.971–1.000	11.7	0.179
CRP	0.762	0.818	0.930	0.794	0.669–0.920	3.44	0.239
ESR	0.763	0.778	32	0.744	0.5568–0.920	3.28	0.29
Esterase	0.944	0. 800	1.5 ^1^	0.872	0.711–1.000	14.4	0.21
Leukocyte count	0.971	0.857	6.74	0.857	0.612–1.000	30.0	0.15
% neutrophils	0.971	0.857	85	0.902	0.726–1.000	30.0	0.15

^1^ Threshold 1.5 for esterase refers to the passage between the first level of the categorical variable (negative) and the second level (POS+); a third level (POS++) was available, and according to the test based on esterase, “POS+” and “POS++” samples were considered septic, while “negative” samples were considered aseptic.

**Table 4:** Correlation matrix depicting the correlation coefficients ρ (with *p*-values) between calprotectin and the tested PJI biomarkers.

	Calprotectin	CRP	ESR	Leukocyte	% Neutrophils
Calprotectin	-	0.49 (0.001)	−0.05 (0.764)	0.69 (<0.001)	0.61 (<0.001)
CRP	-	-	−0.18 (0.270)	0.15 (0.353)	0.16 (0.326)
ESR	-	-	-	0.03 (0.868)	0.35 (0.027)
Leukocyte	-	-	-	-	0.57 (<0.001)
% neutrophils	-	-	-	-	-

## Data Availability

The data presented in this study are openly available in Zenodo DOI: 10.5281/zenodo.7257866 (accessed on November 2022).
